# A functional role of the sky’s polarization pattern for orientation in the greater mouse-eared bat

**DOI:** 10.1038/ncomms5488

**Published:** 2014-07-22

**Authors:** Stefan Greif, Ivailo Borissov, Yossi Yovel, Richard A. Holland

**Affiliations:** 1 School of Biological Sciences, Queen’s University Belfast, 97 Lisburn Road, Belfast BT9 7BL, UK; 2 Sensory Ecology Group, Max Planck Institute for Ornithology, Eberhard-Gwinner-Straße, 82319 Seewiesen, Germany; 3 Department of Zoology, University of Tel Aviv, Klausner Street, PO Box 39040, Tel Aviv 6997801, Israel

## Abstract

Animals can call on a multitude of sensory information to orient and navigate. One such cue is the pattern of polarized light in the sky, which for example can be used by birds as a geographical reference to calibrate other cues in the compass mechanism. Here we demonstrate that the female greater mouse-eared bat (*Myotis myotis*) uses polarization cues at sunset to calibrate a magnetic compass, which is subsequently used for orientation during a homing experiment. This renders bats the only mammal known so far to make use of the polarization pattern in the sky. Although there is currently no clear understanding of how this cue is perceived in this taxon, our observation has general implications for the sensory biology of mammalian vision.

## Abstract (web summary)

Many animals, including insects, birds, fish and reptiles, use polarized light for navigation, but this has not been reported before in mammals. In this study, Greif *et al.* demonstrate that a mammal, the female greater mouse-eared bat, *Myotis myotis*, can also use polarized light for navigation.

## Graphical abstract

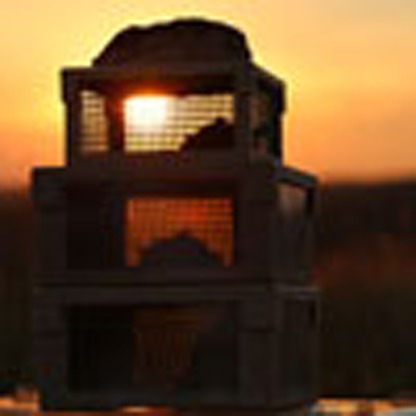

When animals orient and navigate, they rely on a variety of sensory information. This can be the position of the sun or stars, strength and inclination of the Earth’s magnetic field, olfactory cues or the pattern of polarized light in the sky^1^. To achieve the best acuity of orientation and prevent mismatches, these different systems need to be calibrated against one another. Evidence from birds has shown that the pattern of polarized light in the sky is the primary geographical reference against which other cues are calibrated at certain life stages^2^,^3^,^4^,^5^. However, other results indicate that this mechanism might not be straightforward^6^,^7^,^8^,^9^. The use of polarization as an orientation cue is widespread in invertebrates. In vertebrates, birds, fish, reptiles and amphibians have been demonstrated to make use of polarized light, but not mammals^10^,^11^.

Bats use echolocation to orient and navigate. However, this sensory system is only sufficient for a short range of 5–50 m^12^,^13^. For longer range navigation bats must use different sensory input such as vision^14^,^15^. Furthermore, it has been demonstrated that bats can use the Earth’s magnetic field to orient and calibrate it against cues obtained at sunset^16^,^17^. These could be the position of the sun, or as used in birds, the pattern of polarized light. The polarization pattern is strongest at dusk and dawn. At this time a band of maximum polarization stretches 90° east from the position of the sun over the zenith to 90° west of the sun and gets weaker towards and away from the sun) (see natural polarization direction (PN) in Fig. 1a). On the horizon this band is aligned vertically, as is the e-vector of polarization^2^,^10^. To test the hypothesis that bats calibrate a magnetic compass with polarized light cues we conducted translocation experiments with 70 adult female greater mouse-eared bats (*Myotis myotis*). Prior to release, the bats could observe the sky at sunset in experimental boxes with different manipulations of the polarization pattern (natural (PN) versus 90° shifted polarization directions (PS) orientation of the band of maximum polarization).

We hypothesize that bats calibrate their magnetic compass at dusk using the direction of polarization. We predict that, when presented with a vertical polarization 90° left and right to the sunset (corresponding to the direction of the natural band of maximum polarization, Fig. 1a PN) the bats should depart in the home direction when tracked later at the release site. When the box is rotated 90° during sunset (Fig. 1a PS) the bats should also depart ~90° away from the home direction.

## Results

**Figure 1:**
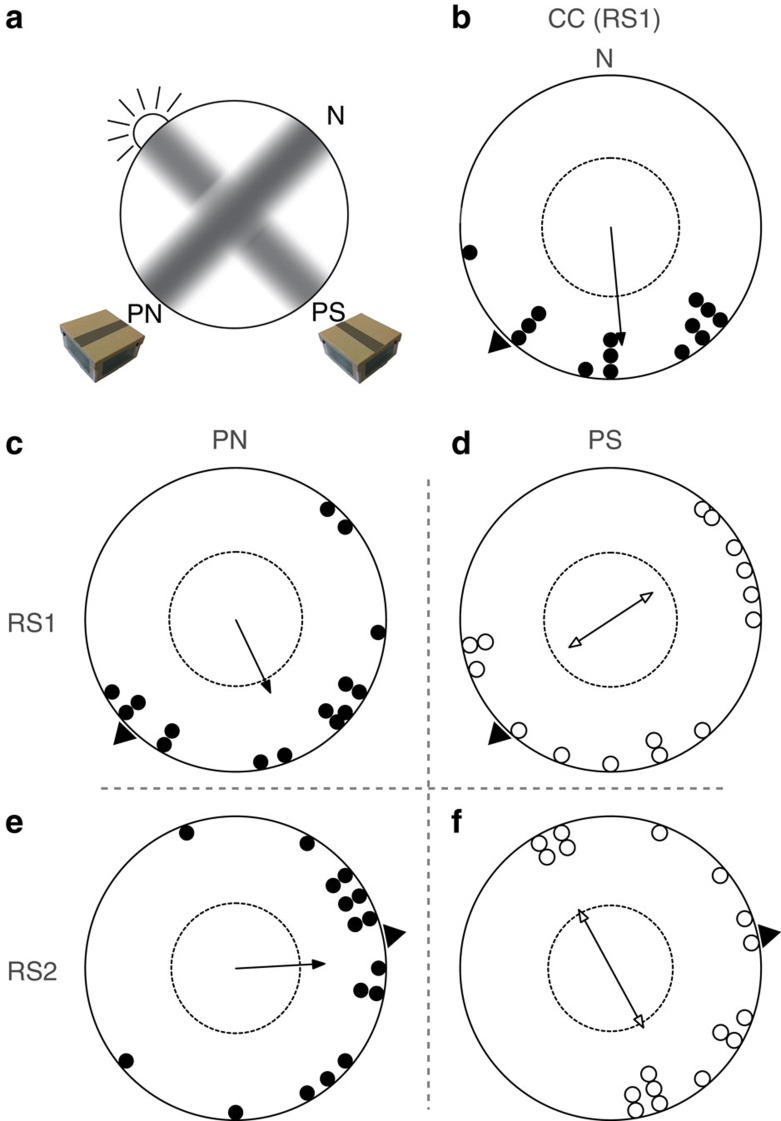
Vanishing bearings of the bats. North (0°) is always at the top of the circles, except for **a**. The dashed circle depicts the Rayleigh significance threshold (*P*=0.05) and the arrows are the mean vector. The axial distribution of PS is shown by a double arrow. The triangle on the outside of the circles indicates the home direction. **a** is a 360° view of the sky with sunset and the experimental conditions. The dark bars represent the band of maximum polarization, mimicked by the filters. PN presents a natural polarization direction pattern, which is shifted 90° in PS. The bars on top of the experimental boxes symbolize the axis of vertical polarization in the windows. **b** are the control bats for RS1 (CC) that were untreated. **c** and **d** show the PN and PS data for release site 1 (RS1) and **e** and **f** for release site 2 (RS2).

For release site one (RS1) the results show that the group with PN had a mean angle significantly different from the home direction (home=224°, confidence interval test: *P*<0.01). However, it was not significantly different from the group of control bats that received no polarization treatment (control (CC), Mardia–Watson–Wheeler test, *W*=3.281, *P*=0.194), which also had a mean angle significantly different from the home direction (confidence interval test: *P*<0.01). Both were significantly oriented (Rayleigh’s test, CC: *Z*=8.376, *P*<0.0001; PN: *Z*=4.221, *P*=0.012, Fig. 1b,c). The group with the PS was significantly different from PN (Mardia–Watson–Wheeler test, *W*=6.936, *P*=0.031), albeit not significantly oriented (Rayleigh’s test, *Z*=1.637, *P*=0.192, Fig. 1d). For release site two (RS2) the mean angle of PN was not significantly different from the home direction (home=80°, confidence interval test: *P*>0.05) and also significantly oriented (Rayleigh’s test, *Z*=5.555, *P*=0.003). We again find that PS and PN are significantly different (Mardia–Watson–Wheeler test, *W*=6.58, *P*=0.037). In addition, the PS group at RS2 shows a significantly axially oriented behaviour (Rayleigh’s test, *Z*=3.335, *P*=0.033, Fig. 1e,f) and was significantly different from home (confidence interval test: *P*<0.01).

## Discussion

These data are the first to support the hypothesis that bats use the polarization pattern in the sky for compass calibration. When the animals were presented with a polarization pattern that equals the natural direction (that is, vertical on the horizon in a North–South axis: PN), they were significantly oriented. At RS1 this is the same as the CC, *albeit* in both cases significantly different from the home direction, which is consistent with previous findings of homing at this site^17^. When treated with a 90° shifted polarization field, PS was significantly different from PN at both sites and at RS2 PS showed a significant axial shift of their vanishing bearing of about 90° in both directions to PN’s mean vector. This behaviour has been described before in experiments manipulating polarization cues and generally is linked to its non-directional nature, meaning that although the polarization pattern can be seen there is no polarity^2^,^18^. In our experiments, the sunset could have been used as a cue to incorporate this information, but apparently bats ignored this and weighed the polarization cue higher. This pattern provides a more reliable calibration point than the sun’s position. Even when the sun’s exact location is obscured by clouds, polarization can still give accurate information. In addition, this precise cue is visible long after the sun has set and is actually most prominent at dusk and dawn, and therefore perceivable when these bats emerge from their caves^2^. Bats are also fairly light sensitive, so a small amount of polarization might be sufficient for their calibration^19^. Recently, experiments have shown that dung beetles can even use the polarization pattern of the moon for orientation^20^,^21^.

Currently, it is unclear how bats could perceive the sky’s polarization pattern. In insects, polarization vision is coupled to clear morphological structures (dichroism) of the photoreceptors^10^,^11^. In vertebrates, oil droplets and double cones have been suggested as a mechanism for birds^9^,^22^. In fish there is also a hypothesized mechanism involving double cones^23^,^24^ and in anchovy the same morphological adaptation of the cone cell receptors is proposed to be involved in polarization vision^25^,^26^ as is in amphibians and reptiles^27^. The only other mammals known so far to be able to perceive polarization are humans. However, there is no known function and the mechanism is still unclear. The alignment of receptors in the macula has been proposed^10^, as has the role of the blue-cone distribution^28^. Little is known about the detailed retinal structure of greater mouse-eared bats, although they do not appear to possess a macula. Some bats however, seem to be sensitive in the ultraviolet range of light^29^,^30^, which also would be beneficial to perceive polarization^31^. Thus, both behavioural and physiological studies are required to elucidate how bats perceive the sky’s polarization pattern and exactly how they use it.

## Methods

### Animal models

Seventy non- or post-lactating adult female greater mouse-eared bats (*M. myotis*) were caught at their home roost (Orlova Chuka Cave, district Ruse) in Northeast Bulgaria, between 12 July and 7 August, 16–22 h before the experimental treatment and were kept at the nearby Siemers Bat Research Station, Tabachka. All experiments were carried out under the licence of the responsible Bulgarian authorities (the Bulgarian Ministry of Environment and Water, and Regional Inspectorate (RIOSV) Ruse, permit #465/29.06.2012).

### Experimental treatment

**Figure 2:**
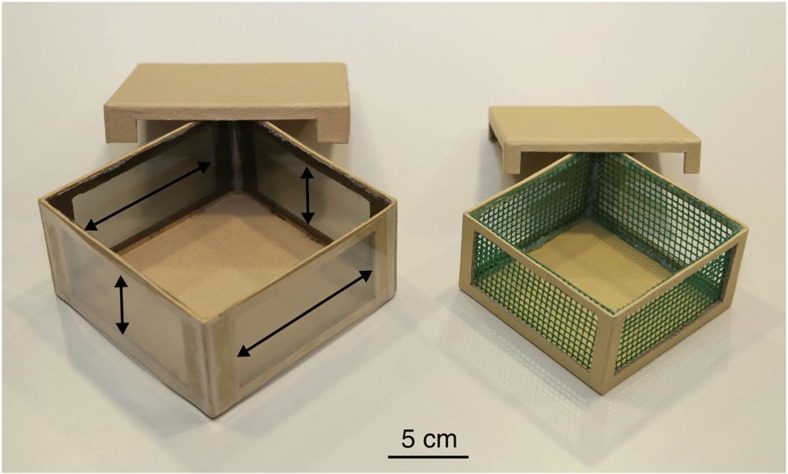
Experimental boxes. On the left is the outer box (polarization box) with two different layers of filters. On the outside is a pseudo-depolarizing filter and on the inside a polarizing filter. The direction of polarization is indicated with arrows. On the right is the inner box (holding box) with its meshed windows.

For the experiments, bats were put in holding cages at a treatment site 1.3 km away from their cave and offered a clear view of the horizon in all directions from 22 min before until 75 min after sunset (when the last visible post-sunset glow had disappeared). All experimental evenings generally had clear sky and always had a visible sunset with only the exceptional cloud coming up. Bats were placed in holding cages which consisted of an inner (120 × 120 × 63 mm) and outer (148 × 148 × 72 mm) cardboard box. The inner holding box had windows (98 × 47 mm) covered with plastic mesh (4 mm square). The outer experimental box also had windows (108 × 52 mm), covered with an outer layer of a pseudo-depolarizing filter (90% depolarization with a 10–15% reduction of light intensity for a range of 400–800 nm)^6^, effectively eliminating natural polarization. The windows further had an inner layer which was a polarizing filter (linear polarizer P500, 3Dlens Corporation Taiwan, ultraviolet block, transmittance: 43%, polarizing: efficiency 99.9% at 380–700 nm)^2^. The polarization direction of the inner filter was either vertically or horizontally oriented, with opposite sides having the same direction (Fig. 2). The boxes were oriented either with the vertically polarized windows 90° away from the sun (in a North–South axis), corresponding to the natural situation (PN), or they were shifted 90° so that now the horizontally polarized windows were oriented North–South (PS) (Fig. 1a). Bats were also kept in a double-wrapped magnetic Helmholtz coil with current antiparallel (resulting in no change of the natural magnetic field), as they served as a control group for another experiment (see Supplementary Data 1 for details of release nights).

### Testing

After the treatment, the bats were translocated to either of two release sites, with RS1 23.6 km north–north–east and RS2 20.4 km south–west–west away from the treatment site. Both sites were flat fields, offering a clear line of sight in all directions. Bats were then fed 20–40 mealworm larvae (*Tenebrio molitor*) and watered *ad libitum*. They were equipped with radio transmitters (BioTrack PicoPip AG379, 0.44 g) and released singly starting at 0100 hours. The bats were tracked from the roof of a car using a radio receiver (AR8200 III, AOR) connected to a five-element Yagi antenna which was mounted on a 4 m pole, thus reaching ~6 m in height. The release direction of the bats was chosen randomly and the only person tracking them was blind to the treatment. Each bat was tracked until the radio signal could no longer be heard and after at least 1 min of silence the direction was noted as the vanishing bearing. Each evening the same number of bats for PN and PS were released with 15 each for RS1 and 16 PN and 17 PS for RS2. For RS1 we added another control group which received no polarization treatment (CC, *n*=14).

### Statistical analysis

Mean bearings and vector lengths of each group were calculated using the Oriana 4.0 circular statistics software package. All groups were tested for significant orientation using the Rayleigh’s test. Tests for significant differences between groups were performed using the Mardia–Watson–Wheeler test. Previous studies have found that in polarization experiments, due to the fact that the cue is axial the treatment group often reacts bimodally^2^,^18^. Thus, we applied a decision rule whereby for each group, if the vector length, *r*_axial_>*r*_unimodal_, data would be analyzed as axial^32^.

## Author contributions

S.G. and R.A.H. designed the study. All authors performed the experiments. S.G. and R.A.H. analyzed the data and wrote the manuscript.

## Additional information

**How to cite this article:** Greif, S. *et al.* A functional role of the sky’s polarization pattern for orientation in the greater mouse-eared bat. *Nat. Commun.* 5:4488 doi: 10.1038/ncomms5488 (2014).

## Supplementary Material

Supplementary Data 1 Raw data of bat releases
